# Gastroesophageal reflux disease and atrial fibrillation: a bidirectional Mendelian randomization study

**DOI:** 10.7150/ijms.95518

**Published:** 2024-05-13

**Authors:** Xiaoli Chen, Aihua Li, Yuanyuan Kuang, Qilin Ma

**Affiliations:** 1 Department of Cardiovascular Medicine, Xiangya Hospital, Central South University, Changsha, Hunan, China.; 2 National Clinical Research Center for Geriatric Disorders, Xiangya Hospital, Changsha, Hunan, China.

**Keywords:** Atrial fibrillation, Causal effect, Mendelian randomization, Gastroesophageal reflux disease, Genetics

## Abstract

**Background:** In observational studies, gastroesophageal reflux disease (GERD) is linked to atrial fibrillation (AF). It is uncertain whether the relationship is due to GERD-induced AF or GERD caused by AF, or confusion with factors related to GERD and AF such as obesity and sleep-disordered breathing. We applied bidirectional Mendelian randomization (MR), in which genetic variations are used as instrumental variables to resolve confounding and reverse causation issues, to determine the causal effect between GERD and AF.

**Methods:** Using summary data from the GERD and AF genome-wide association study (GWAS), a bidirectional MR was performed to estimate the causative impact of GERD on AF risk and AF on GERD risk. The GWAS of GERD meta-analysis comprised 78707 cases and 288734 controls. GWAS summary data for AF, including 45766 AF patients and 191924 controls, were used to genetically predicted AF. The inverse variance weighted (IVW) method was the major MR approach used. MR-PRESSO was implemented to detect heterogeneity and correct the effect of outliers. Weighted median and MR-Egger regression were applied to test heterogeneity and pleiotropy.

**Results:** The genetic instruments of GERD related to increasing the risk of AF, with an OR of 1.339 (95% CI: 1.242-1.444, *p* < 0.001). However, after removing the outlier 8 SNPs, genetically predicted AF was not associated with an elevated risk of GERD (*p* = 0.351).

**Conclusions:** Our result suggested that GERD had a causal effect on AF. However, no evidence was identified that AF elevated the risk of GERD.

## Introduction

Atrial fibrillation (AF) is the clinically prevalent arrhythmia, affecting 0.5% to 2% of the global population 1, 2. AF is the primary risk factor for stroke and heart failure, which increases hospitalization and mortality in AF patients. In addition, the AF incidence is predicted to rise over the next 30 years owing to global aging, which will increase the related socioeconomic burden.

Gastroesophageal reflux disease (GERD) refers to gastro-esophageal reflux-induced esophageal mucosal lesions or troublesome symptoms 3. In observational studies, GERD has been related to AF, however, it is unclear whether GERD increases the risk of AF 4. The observed correlation might be attributable to confounding factors, such as obesity and sleep-disordered breathing, which have been related to both GERD and AF 5-7. Furthermore, the relationship might potentially be due to reverse causation, with AF increasing the risk of GERD rather than vice versa 8. This is reasonable given that the left atrial is in tight physical contact with the lower esophagus, and inflammation in the left atrium may spread to the esophageal adventitia, resulting in esophageal mucosal inflammation. Therefore, further evidence independent of confounding and reverse causation, which is unavoidable in observation research, is required to explore the causal effect between GERD and AF. This evidence would provide information on whether modifying one can reduce the risk of another, which would support clinicians in determining targetable causal factors for GERD or AF.

Since genes are assigned randomly during conception, genetic relationships, unlike observational correlations, are not influenced by confounding and reverse causation. Mendelian randomization (MR) is an effective method for determining the causal effects of modifiable exposure on complex disorders 9. MR utilizes genetic instruments to demonstrate whether a strong relationship between genetic predisposition for exposure and outcome suggests causation. In this study, we applied bidirectional MR to explore the causal relationship between GERD and AF.

## Methods

### Study design description

We conducted a total of two MR analyses to estimate the bidirectional effect between GERD and AF by utilizing summary-level genome-wide association study (GWAS) data. The forward MR analysis served GERD as an exposure and AF as an outcome. While the reverse MR analysis AF as an exposure and GERD as an outcome. In this research, only published summary data were used for re-analysis, hence, no extra ethical approval was needed.

### Selection of genetic instruments for MR analysis

Appropriate genetic instruments for MR analysis were selected from two distinct GWAS summary data. First, single-nucleotide polymorphisms (SNPs) were selected at a threshold of genome-wide significance (*p* < 5 × 10^-8^). Second, SNPs with linkage disequilibrium were excluded (determined by r^2^ < 0.1 and distance located > 1MB). Furthermore, SNPs related to the outcome with *p* < 1 × 10^-5^ were removed. After harmonizing the effect alleles across exposure and outcome, palindromic SNPs with intermediate allele frequencies were excluded. F-statistics were calculated to assess the instrument strength. In general, F > 10 may reduce the bias caused by weak genetic instruments 10, 11.

### Data sources for GERD and AF

The GWAS data related to GERD were provided by ONG *et al.*'s recently published GWAS meta-analysis on GERD 12. The GWAS of GERD meta-analysis comprised 2 cohorts of European ancestry with 78707 cases and 288734 controls (Table 1). GERD is diagnosed according to the ICD-10 and ICD-9, which are based on the subject symptoms, operative procedures, and the use of GERD medication. This study identified 88 risk loci association with GERD as genetic instruments in Multi-Trait Analysis of GWAS that combine GERD with BMI, major depressive disorder, and education attainment (*p* < 5.0×10^-8^) 12. These SNPs explained 3.92% of the variation in GERD, and the F-statistics was 124 suggesting that the instruments had a substantial ability to predict GERD.

We obtained GWAS summary data for AF from the FinnGen consortium (released on May 11, 2023) to reduce potential bias due to sample overlap and demographic stratification. The FinnGen consortium includes aggregated genetic data and illness trajectories from up to 377,277 Europeans, 16 962 023 variations, and 2272 disease endpoints 13. In the FinnGen consortium, the summary data for AF included 45766 AF patients and 191924 controls. Atrial fibrillation was mainly diagnosed according to ICD-9 or ICD-10.

### Statistical analyses

For MR analysis, 3 fundamental assumptions should be satisfied: (i) the genetic instruments are powerfully linked to the exposure; (ii) the genetic tools are independent of other confounders; (iii) the genetic tools are relevant to the outcome solely via the exposure 14. The causal effect between GERD and AF was estimated by using bidirectional MR. We applied several MR methods to estimate the effect after harmonizing the effect alleles across the summary statistic, including inverse-variance weighted (IVW), weighted median, and MR-Egger regression. Multiple methods were applied since the underlying assumptions for horizontal pleiotropy differed 15. The IVW estimates the effect by performing a meta-analysis of the Wald ratio for single SNPs, which was applied as the major outcome. The approach presumes that all SNPs are effective instruments and that instruments of exposure cannot affect the outcome through other alternative pathways. The weighted median estimator calculates the median effect of SNPs, with < 50% invalid SNPs allowed 16. The MR-Egger regression allows for the appearance of horizontal pleiotropy across SNPs, with a loosened criterion. The IVW estimation are supplement by weighted median and MR-Egger methods, since they can produce more reliable estimates under a broader range of scenario but are less efficient and have larger confidence intervals 15. If the estimations of these methods were contradictory in our study, a tightening instrument *p*-value threshold was established 17. MR-Pleiotropy Residual Sum and Outlier methods (MR-PRESSO) were implemented to detect and correct the effect of outliers and produce a causal estimation that was robust to heterogeneity 18. Furthermore, we applied Radial regression of MR (Radial MR) to retrieve outlier SNPs, as a supplementary method for MR-PRESSO 19.

### Sensitivity analyses

The Cochran's Q test in the IVW method was used to assess the heterogeneity of results (*p* < 0.05 was defined as the existence of heterogeneity). The intercept of MR-Egger regression was an indication of directional pleiotropy (The existence of directional pleiotropy was defined as *p* < 0.05) 20. Leave-one-out was conducted to assess whether a single SNP was driving or biasing the MR estimate. Once we detected heterogeneity or horizontal pleiotropy, we recalculated IVW, weighted median and MR-Egger estimates after eliminating outlier SNPs determined by MR-PRESSO or Radial MR. All analyses were performed in R (version 4.2.1) with R packages 'TwoSample MR', 'MR-PRESSO', and 'Radial MR'.

## Results

### Effect of GERD on AF risk

**Figure 1:**
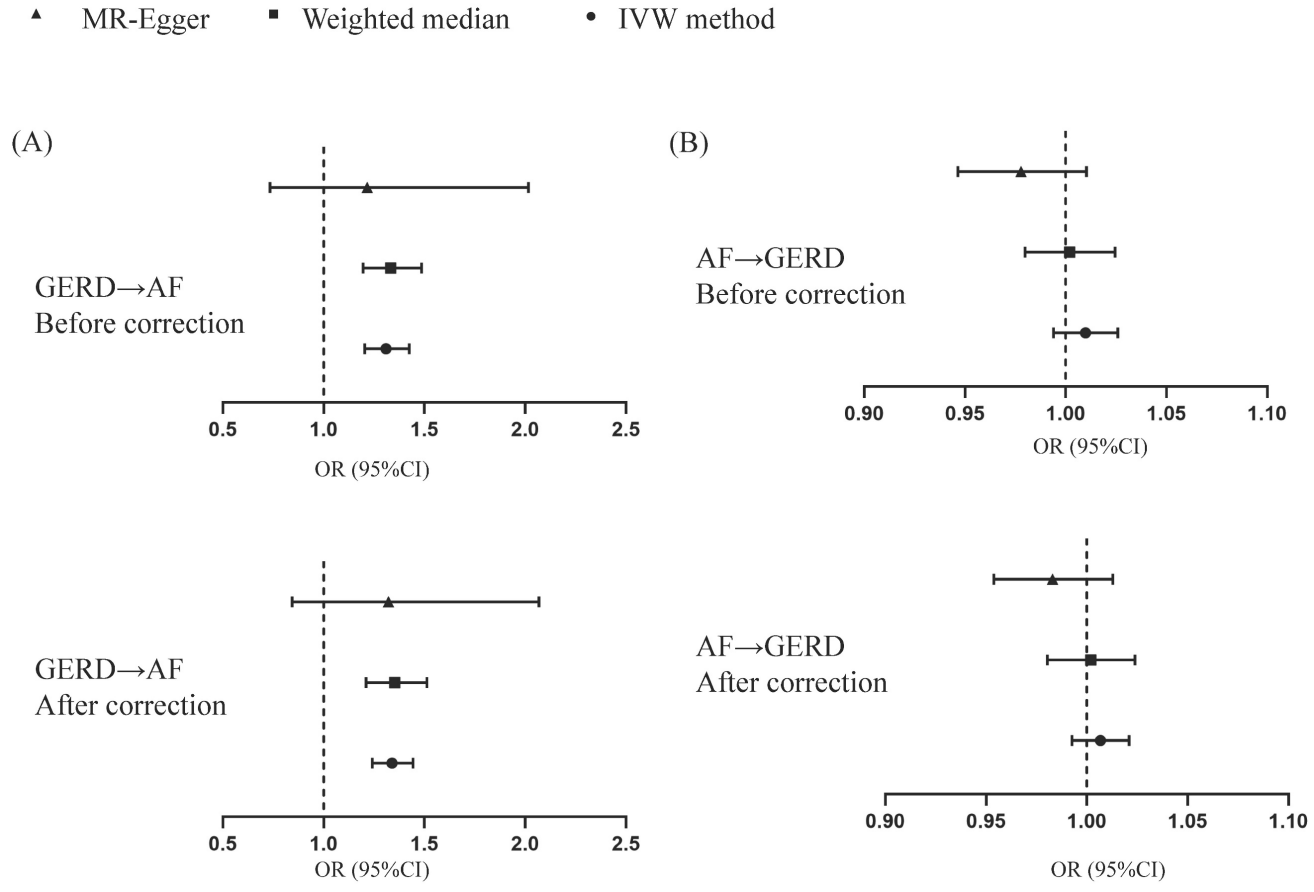
Causal estimates for effect of GERD on AF and vice versa. MR, Mendelian randomization; GERD, Gastroesophageal reflux disease; AF, Atrial fibrillation.

**Figure 2:**
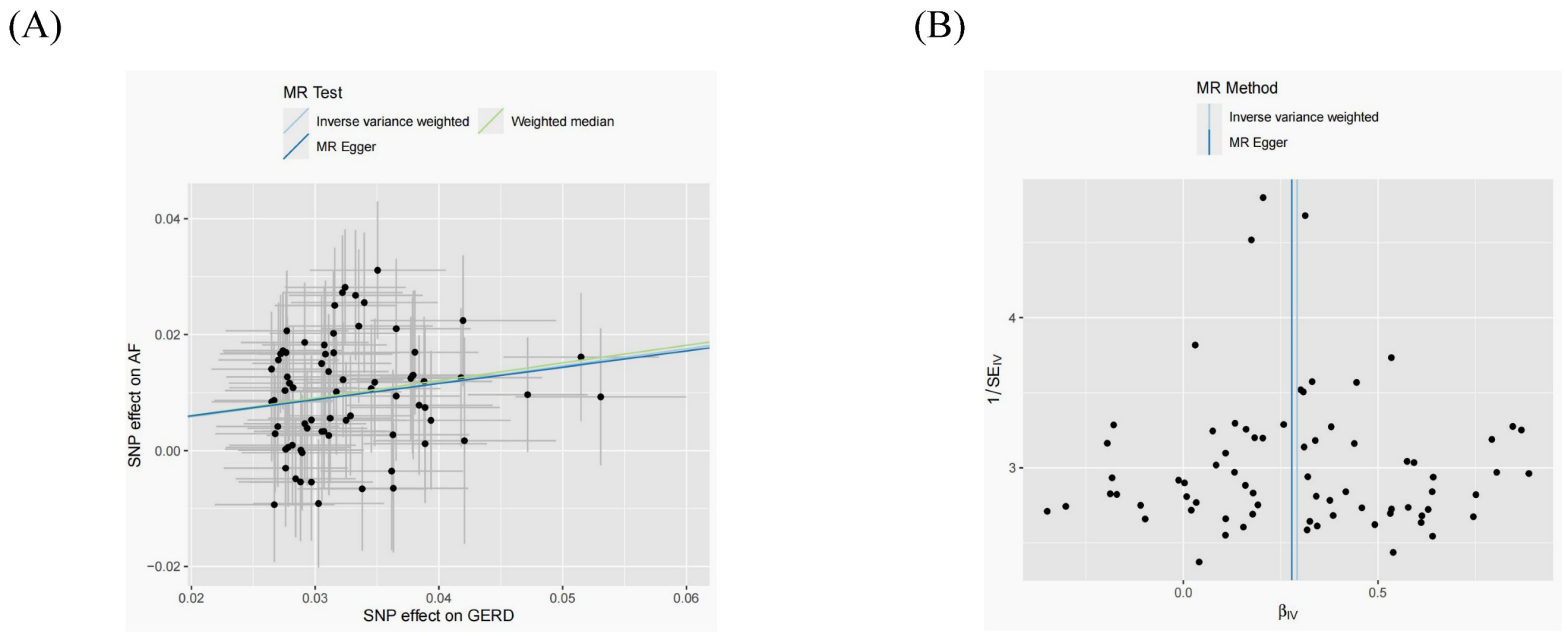
Scatter plots and funnel plots for effects of GERD on AF MR (A, B). MR, Mendelian randomization; GERD, Gastroesophageal reflux disease; AF, Atrial fibrillation.

**Figure 3:**
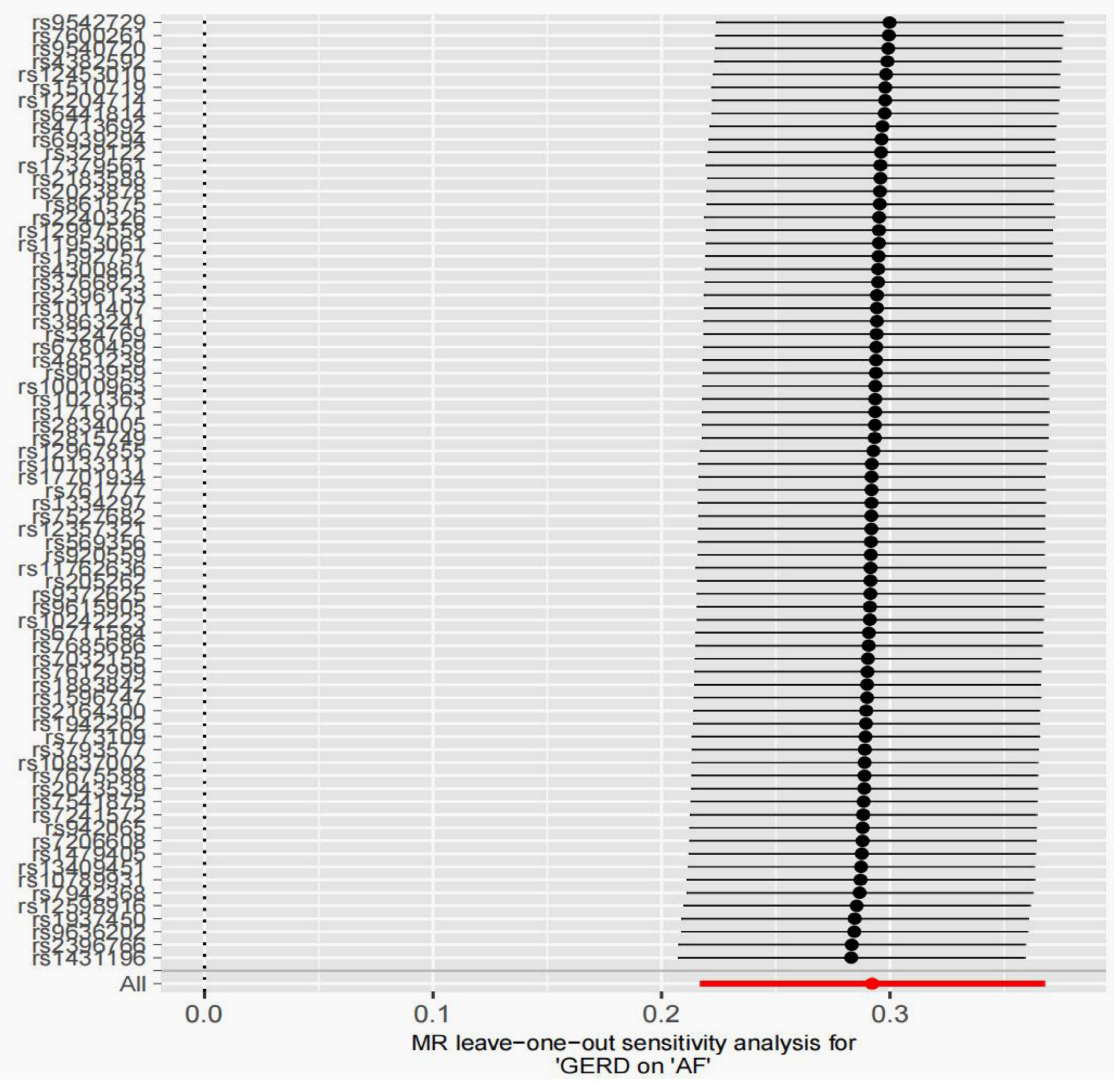
MR leave-one-out sensitivity analysis for AF on GERD after removing outliers. MR, Mendelian randomization; GERD, Gastroesophageal reflux disease; AF, Atrial fibrillation.

After removing 3 palindromic SNPs with intermediate allele frequencies (rs2358016, rs9517313, and rs957345), 2 SNPs associated with outcome (rs9373363 and rs9940128) and 2 SNPs missing in exposure from 88 SNPs for GERD in the GWAS meta-analysis, the remaining 81 SNPs were used to the genetic instrument for GERD (Table S1). The genetic susceptibility to GERD has a potential causal effect on AF risk (OR = 1.310; 95%CI: 1.204-1.425, *p* < 0.001) (Figure 1A, Table 2). Furthermore, the weighted median approach showed similar results (OR = 1.333, 95%CI: 1.196-1.486, *p* < 0.001). No significant statistical differences were noted in the MR-Egger method (OR: 1.216, 95% CI: 0.734-2.016, *p* = 0.450). Heterogeneity was noted, the *p* value calculated by Cochran Q-text was < 0.05. In addition, the global heterogeneity test of MR-PRESSO showed the same result with *p-*value < 0.05. After removing 8 outlier SNPs by MR-PRESSO and Radial MR methods, the MR methods were re-used to evaluate the risk of developing AF in the context of GERD. With the IVW method, GERD increased the risk of AF (OR 1.339, 95% CI: 1.242-1.444, *p* < 0.001) (Figure 1A, Table 2). A similar result was obtained from the weighted median methods (OR 1.353, 95% CI: 1.210-1.513, *p* < 0.001) and MR-Egger approach, although no statistically significant was observed in MR-Egger approach (OR: 1.321, 95% CI: 0.844-2.067, *p* = 0.227). In MR analyses of GERD on AF, the *p-*value of the MR-Egger intercept test was >0.05, indicating that no significant pleiotropy was detected. Figure 2 showed the MR regression slopes. Leave-one-out analyses showed that none of SNP significantly biased in the overall effect of GERD on AF (Figure 3). The funnel plot showed symmetry, excluding the pleiotropic effect (Figure 2).

### Effect of AF on GERD risk

**Figure 4:**
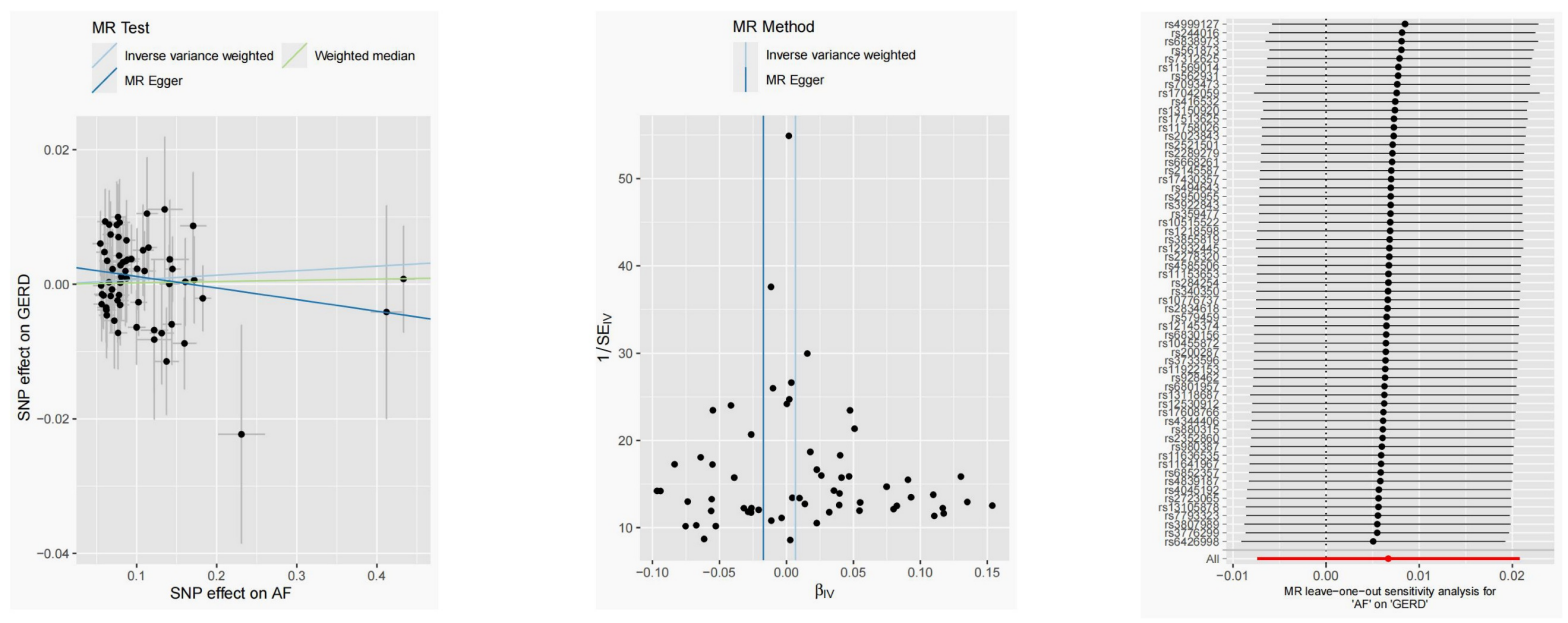
Causal estimates for the effect of AF on GERD. (A-B) Scatter plots and funnel plots for effects of AF on GERD. (C) MR leave-one-out sensitivity analysis for AF on GERD after removing outliers.

After harmonization, 69 SNPs were used as genetic instruments for AF (Table S2). These SNPs explained 26.9% of the variance in AF, and the F-statistic = 1270 suggesting adequate instruments strength. Using the 69 AF-related SNPs, no statistical evidence for a potential causal effect of AF on GERD was noted (OR = 1.010; 95%CI: 0.994-1.026, *p* = 0.228) (Figure 1B, Table 2). Meanwhile, the weighted median (*p* = 0.866) and MR-Egger regression (*p* = 0.183) methods yielded similar risk estimates. However, the *p*-value of the Cochran Q-test and the global test *p-*value of MR-PRESSO were both < 0.05, indicating the presented of heterogeneity in IVW. The MR-PRESSO and Radial MR approach were used to remove outlier SNPs and the MR methods were re-used to estimate the effect of AF on GERD. The results suggested that AF was not linked to an increased risk of GERD (OR = 0.983; 95%CI: 0.954-1.013, *p* = 0.351) (Figure 2B, Table 2). Similar results were gained in the weighted median and MR-Egger regression (*p* > 0.05). No directional pleiotropy was observed in the MR-Egger intercept test (Figure 4). The leave-one-out analysis revealed that the effect of AF on GERD was not significantly driven by a single SNP (Figure 4). Furthermore, the funnel plot showed symmetry (Figure 4).

## Discussion

In this study, we conducted bidirectional two-sample MR analyses to assess the causal effect between GERD and AF. Our findings show that GERD aggravates the risk of developing AF, however, the direct causal effect of AF on GERD remains uncertain.

The correlation between GERD and AF has been reported in some studies 21-24. The existence of GERD may raise the incidence of AF by approximately 39% in a retrospective and small-scale population study 22. Similar results were obtained from the Taiwan National Health Insurance database. In this prospective and nationwide population-based cohort, GERD was related to an elevated risk of AF (HR 1.31; 95%CI: 1.06-16.1) 23. However, inconsistent results were observed in several studies 21, 25. Bunch TJ *et al.*'s study demonstrated that there was no association between GERD on the risk of AF after adjusting of confounders, by using a self-reported questionnaire to evaluate the GERD frequency of 5288 Olmsted county inhabitants 21. Furthermore, in a recent meta-analysis consisting of 4 studies (29671 controls and 83882 GERD cases), the outcomes showed that GERD had no statistical difference in the occurrence of AF, with an RR of 1.06 (95% CI: 0.86-1.31) and high heterogeneity (*p* = 0.004; I^2^ = 77.6%).^26^. Most cross-sectional epidemiological research failed to elucidate causation with a hazy temporal order. Residual confounding and reverse causation were hard to avoid in observational studies, so causal inferences cannot be confidently revealed. In this study, we used MR methods to overcome the impact of confounding and evaluate the causal effect of GERD on AF. The risk of developing AF elevated by 34% in the presence of GERD (OR 1.34, 95% CI: 1.24-1.44, *p* < 0.001).

The potential mechanism for how GERD may raise AF risk remains unclear. The dysfunction of the autonomic nervous system (ANS) is essential for the initiation and maintenance of AF. Previous research demonstrated that the balance of sympathovagal was altered by electrical, chemical, and mechanical stimulation of the esophagus 27. The stimulation of esophageal seems to augment respiratory-drive cardiac vagal-afferent regulation and lowering sympathetic regulation 27. Acid reflux stimulates the vagus nerve to regulate the distal esophagus through the esophageal plexus, where it induces cardiac autonomic reaction to esophageal sensory signals and regulates peristalsis and secretory function 27, 28. In addition, distal esophageal injury can further affect vagal nerve responses, notably nerve sensitization of afferent routes 29. Acid reflux creates a local inflammation that can affect the autonomic innervations of esophageal mucosa as well as permeate the esophageal wall to stimulate the nearby vagal nerves 29. Furthermore, several studies have shown that gastric acid suppression treatment with proton pump inhibitors (PPIs) may support alleviated AF symptoms and also promote conversion AF to sinus rhythm, indirectly implying a causal relationship 30, 31. In 18 patients with GERD and AF treated with PPIs, the epigastric pain and inflammation were alleviated, and the onset of AF either completely stopped or reduced in frequency 31. So, we further analyzed the effect of proton pump PPIs on AF by MR. SNPs related to the use of omeprazole, a commonly used PPIs, were used as instrumental variables. The result of inverse-variance weighted (IVW) suggested no potential causal effect of omeprazole on AF (OR = 3.79; 95%CI: 0.78-18.38, *p* = 0.097, Figure S1). Furthermore, the weighted median and MR-Egger approaches showed similar results (*p* > 0.05, Figure S1)). These results prompted us to speculate that the alleviation of atrial fibrillation symptoms by PPIs in observational studies may be mediated by improving GERD.

The theoretical potential of reverse causation was characterized, in which the relationship between GERD and AF is driven by AF rather than GERD 24, 32. In a longitudinal case-control study, patients were divided into two groups based on AF and sinus rhythm and followed up for 3 years. The results showed a higher prevalence of GERD in the AF group than in the sinus rhythm, indicating that AF is a possible cause of GERD (HR of 1.37, 95% CI of 1.16‐1.57, P = 0.009) 32. Due to the adjacent anatomical relationship between the esophagus and left atrium potentially leading to vagal hyperstimulation and local inflammation, possible mechanisms have been proposed. The enlarged and fibrotic left atrium may stimulate the adjacent esophagus. In addition, inflammation is related to the pathophysiology of both AF and GERD. Inflammatory factors comprising leukocytes and interleukin 6 (IL-6) and IL-8 play an essential role in the development of GERD 24. However, Coutinho EL's study reported that the incidence of GERD in paroxysmal AF was low and comparable to the local population 33. Our study suggested no evidence of a causal effect of AF on GERD risk. The possible relationship between AF and GERD may be related to the treatment of AF. Nakaji *et al.* used a multicenter questionnaire survey to explore the effect of common cardiac medications on the occurrence of GERD. They determined that both warfarin and calcium channel blockers were independent risk factors for symptomatic GERD 34. Furthermore, a clinical trial reported that dabigatran, a new oral anticoagulant, induced upper gastrointestinal nonbleeding adverse effects in 16.9% compared to 9.4% in the warfarin group with an RR of 1.81 35. Compared to dyspepsia or dyskinesia alone, the most common adverse event was GERD, with symptoms independent of dose. The ablation technique may cause esophageal thermal damage, with severity ranging from erythema to esophagitis, ulceration, necrosis, and eventually fistula development 36. In addition, substantial acid reflux is observed during radiofrequency catheter ablation targeting AF 37. These studies suggested that the risk of AF on GERD may be attributed to confounding factors such as medication and surgical treatment.

### Strengths and limitations

A major advantage of this study is that, for the first time, we used a bidirectional 2-sample MR method to investigate the causative effect of GERD on the development of AF and vice versa. In comparison to observational research, this method is less affected by confounding and reverse causality. Furthermore, our study overcomes the constraints of observational studies, and establishes GERD as a risk for AF. There are several limitations to this study: First, the total elimination of potential horizontal pleiotropy is difficult 15. However, there was no indication of pleiotropic effect in MR-Egger, and similar results were reported in sensitivity analyses and leave-one-out. Secondly, the findings of this study are based on data from patients of European ancestry, therefore, its applicability to other ethnicities is restricted.

## Conclusions

Using a bidirectional MR method, we overcame the inherent constraints of observational studies and demonstrated that GERD elevated the risk of AF, but there was no evidence that AF raised the risk of GERD.

## Supplementary Material

Supplementary figure and tables.

## Figures and Tables

**Table 1:** Details of the datasets used in this Mendelian randomization study

Exposure/Outcome(s)	Consortium or cohort	Participants		Web source
		Cases	controls	
GERD	UKBB GERD GWAS	75 720	278 565	https://www.ebi.ac.uk/gwas/studies/GCST90000514
	Australian QSKIN GERD GWAS	2987	10 169	
AF	FinnGen	45766	191924	https://www.finngen.fi/en

**Table 2:** Causal estimates from the summary level data-based MR

Exposure	Outcome		No. of SNPs	Cochran's Q statistics for heterogeneity	MR-Egger pleiotropy test P value	MR method	Beta	Standard error	P-value	OR (95%CI)
The forward MR analyses										
GERD		AF	81	0.018	0.772	Inverse variance weighted	0.270	0.043	<0.001	1.310 (1.204-1.425)
						Weighted median	0.287	0.055	<0.001	1.333 (1.196-1.486)
						MR Egger	0.196	0.258	0.450	1.216 (0.734-2.016)
GERD		AF	73	0.932	0.952	Inverse variance weighted	0.292	0.038	<0.001	1.339 (1.242-1.444)
						Weighted median	0.302	0.057	<0.001	1.353 (1.210-1.513)
						MR Egger	0.278	0.229	0.227	1.321 (0.844-2.068)
The reverse MR analyses										
AF		GERD	69	0.032	0.032	Inverse variance weighted	0.010	0.008	0.228	1.010 (0.994-1.026)
						Weighted median	0.002	0.011	0.866	1.002 (0.980-1.024)
						MR Egger	-0.022	0.017	0.183	0.978 (0.946-1.010)
AF		GERD	61	0.890	0.082	Inverse variance weighted	0.007	0.007	0.351	0.983 (0.954-1.013)
						Weighted median	0.002	0.011	0.864	1.002 (0.980-1.024)
						MR Egger	-0.017	0.015	0.265	1.007 (0.993-1.021)
										
