# Supplementary material for: Gastroesophageal reflux disease and atrial fibrillation: a bidirectional Mendelian randomization study
Source: Int J Med Sci. 2024 May 13;21(7):1321–8. doi: 10.7150/ijms.95518 (PMC11134582; doi:10.7150/ijms.95518)
Supplement: Supplementary file 1 — Supplementary figure and tables. [file ijmsv21p1321s1.pdf]

Supplement Figure

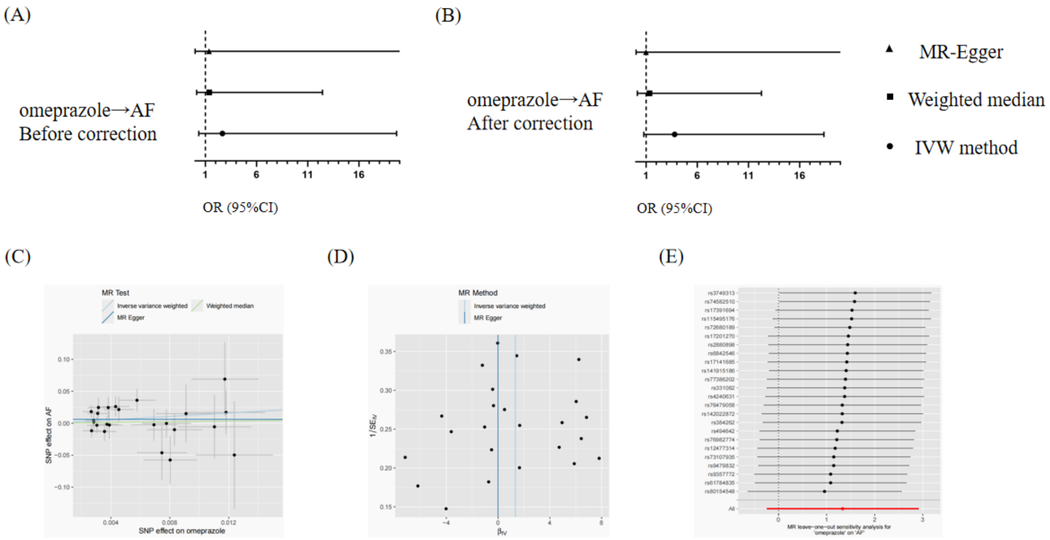

Figure S1: Causal estimates for the effect of omeprazole on AF. (A-B) the OR value of omeprazole on AF. (C-D) Scatter plots and funnel plots for effects of omeprazole on AF. (E) MR leave-one-out sensitivity analysis for omeprazole on AF after removing outliers.

Supplementary Table S1: Genetic data for the MR on the effect of GERD on AF

SNP	effect_allele	other_allele	eaf.exposure	beta.exposure	se.exposure	pval.exposure
rs10010963	C	T	0.38	0.027	0.005	4.92E-08
rs1011407	A	G	0.88	0.042	0.007	1.09E-08
rs10133111	G	A	0.84	-0.042	0.007	1.35E-10
rs1021363	A	G	0.36	0.031	0.005	5.10E-10
rs10242223	A	G	0.33	0.028	0.005	3.79E-08
rs10789931	C	T	0.88	-0.042	0.007	2.09E-08
rs10837002	C	G	0.65	-0.028	0.005	4.03E-08
rs11645288	G	A	0.81	-0.034	0.006	2.78E-08
rs11762636	C	A	0.82	0.051	0.006	1.88E-16
rs11953061	C	T	0.66	-0.028	0.005	3.10E-08
rs12204714	C	T	0.37	0.029	0.005	7.92E-09
rs12357321	G	A	0.69	-0.032	0.005	1.33E-09
rs12453010	C	T	0.61	-0.030	0.005	1.75E-09
rs12598916	C	G	0.73	0.033	0.005	6.87E-10
rs12967855	A	G	0.33	0.037	0.005	1.09E-12
rs12997558	G	A	0.64	-0.028	0.005	3.04E-08
rs13107325	C	T	0.93	-0.070	0.009	2.20E-14
rs1334297	G	A	0.27	0.039	0.005	1.14E-12
rs13409451	A	G	0.61	0.028	0.005	1.93E-08
rs1431196	A	G	0.57	-0.032	0.005	2.65E-11
rs1479405	C	T	0.68	-0.031	0.005	9.85E-10
rs1510719	T	C	0.62	0.039	0.005	3.84E-15
rs1592757	G	C	0.64	-0.031	0.005	6.00E-10
rs1596747	A	G	0.51	-0.031	0.005	1.00E-10
rs1716171	C	T	0.21	-0.038	0.006	7.82E-11
rs17379561	A	T	0.86	-0.053	0.007	1.08E-14
rs17701934	T	C	0.56	0.026	0.005	4.60E-08
rs1883842	T	G	0.72	-0.031	0.005	9.27E-09
rs1937450	T	G	0.46	-0.032	0.005	7.07E-11
rs1942262	G	A	0.71	-0.032	0.005	2.60E-09
rs2016933	C	G	0.27	0.031	0.005	1.04E-08
rs2023878	C	T	0.81	0.036	0.006	3.04E-09
rs2043539	G	A	0.58	-0.027	0.005	2.24E-08
rs205262	A	G	0.73	-0.035	0.005	1.38E-10
rs215614	G	A	0.37	0.033	0.005	4.08E-11
rs2164300	C	T	0.48	0.026	0.005	4.13E-08
rs2183588	A	G	0.35	-0.029	0.005	1.22E-08
rs2240326	G	A	0.53	0.047	0.005	1.13E-22
rs2396133	A	G	0.52	-0.029	0.005	1.11E-09
rs2396766	G	A	0.53	-0.032	0.005	2.33E-11
rs2734839	C	T	0.39	0.028	0.005	8.79E-09
rs2782641	G	A	0.39	-0.027	0.005	4.33E-08
rs2815749	A	G	0.20	-0.039	0.006	1.07E-10
rs2834005	T	C	0.69	-0.030	0.005	9.42E-09
rs2838771	G	C	0.35	0.028	0.005	2.91E-08
rs324769	C	T	0.55	0.027	0.005	3.05E-08
rs329122	G	A	0.58	0.029	0.005	3.05E-09
rs3766823	G	A	0.83	-0.039	0.006	7.09E-10
rs3793577	A	G	0.46	-0.027	0.005	2.49E-08
rs3863241	C	T	0.47	-0.032	0.005	1.49E-11
rs4300861	C	T	0.62	-0.031	0.005	5.43E-10
rs4382592	T	G	0.30	0.030	0.005	8.20E-09
rs4713692	C	T	0.63	0.028	0.005	3.07E-08
rs4851239	C	T	0.62	0.033	0.005	3.24E-11
rs569356	A	G	0.86	0.038	0.007	4.07E-08
rs6441814	G	A	0.53	0.028	0.005	3.86E-09

rs6711584	G	A	0.55	-0.032	0.005	2.66E-11
rs6780459	A	T	0.25	-0.031	0.006	3.14E-08
rs6939294	C	T	0.77	0.036	0.006	2.71E-10
rs7032155	C	A	0.41	-0.028	0.005	1.63E-08
rs7206608	C	G	0.68	-0.029	0.005	1.46E-08
rs7241572	G	A	0.79	-0.037	0.006	9.49E-10
rs7527682	A	G	0.46	0.027	0.005	3.13E-08
rs7541875	A	G	0.57	-0.027	0.005	1.61E-08
rs7600261	C	T	0.69	-0.034	0.005	9.47E-11
rs7612999	G	A	0.75	-0.031	0.006	4.90E-08
rs761777	A	G	0.75	-0.035	0.006	4.71E-10
rs7675588	C	A	0.21	0.034	0.006	1.80E-08
rs7685686	A	G	0.58	0.028	0.005	1.14E-08
rs773109	G	A	0.66	0.038	0.005	8.71E-14
rs7942368	C	T	0.79	0.034	0.006	9.54E-09
rs861575	T	C	0.58	-0.028	0.005	1.63E-08
rs903678	G	A	0.66	-0.028	0.005	4.89E-08
rs903959	T	A	0.60	-0.029	0.005	2.99E-09
rs920559	C	G	0.85	-0.038	0.007	2.06E-08
rs9372625	G	A	0.62	0.038	0.005	2.62E-14
rs942065	G	A	0.37	-0.031	0.005	8.45E-10
rs9540720	A	G	0.52	-0.027	0.005	3.01E-08
rs9542729	C	G	0.80	0.036	0.006	1.41E-09
rs9615905	C	T	0.54	-0.028	0.005	1.21E-08
rs9636202	G	A	0.73	0.035	0.005	1.51E-10

eaf.outcome	beta.outcome	se.outcome	pval.outcome
0.335	0.004	0.010	0.688
0.918	0.002	0.018	0.923
0.781	-0.013	0.012	0.288
0.274	0.006	0.011	0.612
0.318	0.011	0.011	0.302
0.738	-0.022	0.011	0.046
0.681	-0.017	0.010	0.108
0.758	0.017	0.011	0.135
0.724	0.016	0.011	0.143
0.623	-0.001	0.010	0.927
0.368	-0.005	0.010	0.596
0.702	-0.010	0.011	0.347
0.630	0.005	0.010	0.591
0.740	0.027	0.011	0.017
0.266	0.009	0.011	0.397
0.644	-0.001	0.010	0.956
0.986	0.103	0.042	0.014
0.270	0.012	0.011	0.280
0.665	0.021	0.010	0.046
0.586	-0.028	0.010	0.005
0.701	-0.020	0.011	0.059
0.637	0.001	0.010	0.907
0.652	-0.003	0.010	0.799
0.501	-0.014	0.010	0.165
0.212	-0.008	0.012	0.514
0.776	-0.009	0.012	0.429
0.635	0.008	0.010	0.411
0.815	-0.017	0.013	0.189
0.432	-0.025	0.010	0.011
0.762	-0.017	0.012	0.145
0.241	-0.019	0.011	0.099
0.739	0.003	0.011	0.806
0.633	-0.017	0.010	0.101
0.719	-0.012	0.011	0.281
0.283	0.032	0.011	0.004
0.531	0.014	0.010	0.152
0.416	0.000	0.010	0.993
0.457	0.010	0.010	0.325
0.545	-0.004	0.010	0.696
0.544	-0.027	0.010	0.006
0.476	0.031	0.010	0.002
0.396	0.030	0.010	0.003
0.141	-0.007	0.014	0.598
0.728	-0.005	0.011	0.632
0.290	-0.017	0.011	0.109
0.387	0.003	0.010	0.772
0.579	0.000	0.010	0.971
0.781	-0.005	0.012	0.661
0.490	-0.016	0.010	0.114
0.416	-0.005	0.010	0.599
0.563	-0.003	0.010	0.736
0.274	-0.009	0.011	0.408
0.613	-0.003	0.010	0.763
0.652	0.006	0.010	0.558
0.870	0.013	0.015	0.369
0.610	-0.005	0.010	0.630

0.544	-0.012	0.010	0.214
0.212	-0.003	0.012	0.782
0.847	-0.004	0.014	0.795
0.374	-0.013	0.010	0.210
0.646	-0.019	0.010	0.069
0.784	-0.021	0.012	0.080
0.378	0.009	0.010	0.390
0.616	-0.017	0.010	0.087
0.697	0.007	0.011	0.537
0.768	-0.015	0.012	0.197
0.726	-0.011	0.011	0.330
0.165	0.021	0.013	0.103
0.476	0.012	0.010	0.236
0.697	0.017	0.011	0.112
0.791	0.026	0.012	0.034
0.527	0.000	0.010	0.981
0.705	-0.032	0.011	0.003
0.622	-0.005	0.010	0.646
0.843	-0.013	0.013	0.337
0.681	0.012	0.011	0.237
0.374	-0.018	0.010	0.072
0.467	0.009	0.010	0.343
0.727	-0.006	0.011	0.557
0.564	-0.010	0.010	0.295
0.778	0.031	0.012	0.009

Supplementary Table S2: Genetic data for the MR on the effect of AF on GERD

SNP	effect_allele	other_allele	eaf.exposure	beta.exposure	se.exposure
rs10455872	G	A	0.046	0.141	0.023
rs10515522	C	T	0.194	0.078	0.012
rs10738610	C	A	0.418	0.070	0.010
rs10776737	G	A	0.240	0.081	0.011
rs11153653	C	T	0.341	0.065	0.010
rs11569014	A	G	0.028	0.231	0.029
rs11636535	C	T	0.293	0.087	0.011
rs11641967	T	G	0.156	0.075	0.013
rs11758026	C	T	0.285	-0.079	0.011
rs11922153	G	A	0.110	0.086	0.016
rs12145374	C	A	0.166	-0.110	0.013
rs1218598	A	G	0.811	-0.141	0.012
rs12530912	G	T	0.251	-0.088	0.011
rs12932445	C	T	0.232	0.172	0.011
rs13105878	A	C	0.107	-0.170	0.016
rs13118687	A	G	0.483	-0.145	0.010
rs13150920	T	C	0.538	0.063	0.010
rs17042059	A	G	0.143	0.433	0.013
rs17430357	T	A	0.162	0.076	0.013
rs17513625	A	G	0.064	0.412	0.019
rs17608766	C	T	0.176	0.108	0.013
rs200287	T	A	0.865	-0.083	0.014
rs2023843	T	C	0.904	0.122	0.017
rs2145587	A	G	0.376	0.056	0.010
rs2278320	C	T	0.194	0.069	0.012
rs2289279	C	T	0.173	-0.072	0.013
rs2352860	C	G	0.055	-0.135	0.022
rs244016	G	A	0.762	-0.144	0.011
rs2521501	T	A	0.266	0.062	0.011
rs2723065	G	A	0.342	-0.077	0.010
rs2834618	G	T	0.114	-0.086	0.016
rs284254	G	A	0.131	0.079	0.015
rs2950955	A	C	0.332	0.059	0.010
rs295149	T	C	0.369	-0.056	0.010
rs340350	A	G	0.864	-0.087	0.014
rs359477	C	T	0.467	-0.057	0.010
rs3733596	G	C	0.767	-0.080	0.011
rs3776299	A	G	0.505	0.061	0.010
rs3807989	G	A	0.571	0.115	0.010
rs3816470	G	A	0.566	-0.054	0.010
rs3855819	G	C	0.161	-0.161	0.014
rs3922843	G	A	0.771	-0.067	0.012
rs4045192	C	T	0.498	-0.067	0.010
rs416532	G	T	0.569	-0.102	0.010
rs4344406	G	C	0.391	-0.060	0.010
rs4585506	T	C	0.392	-0.056	0.010
rs4615152	C	T	0.719	0.067	0.011
rs4839187	T	C	0.184	0.079	0.013
rs494643	A	G	0.749	-0.062	0.011
rs4999127	A	G	0.863	0.160	0.015
rs561873	A	G	0.128	-0.137	0.015
rs562931	A	C	0.730	-0.077	0.011
rs579459	T	C	0.779	-0.070	0.012
rs6426998	T	C	0.543	-0.077	0.010
rs653178	T	C	0.587	-0.055	0.010
rs6668261	T	C	0.055	0.122	0.022

rs6801957	C	T	0.548	0.063	0.010
rs6830156	G	T	0.822	-0.100	0.013
rs6838973	T	C	0.498	-0.183	0.010
rs6852357	T	C	0.164	0.113	0.013
rs7093473	C	T	0.122	0.131	0.015
rs7093996	G	T	0.703	0.062	0.011
rs7312625	A	G	0.695	0.100	0.011
rs7699969	A	G	0.499	-0.062	0.010
rs7731963	C	A	0.607	-0.057	0.010
rs7793323	C	T	0.328	0.066	0.010
rs880315	C	T	0.412	0.093	0.010
rs928462	C	T	0.187	-0.078	0.013
rs980387	T	C	0.468	0.055	0.010

pval.exposure	eaf.outcome	beta.outcome	se.outcome	pval.outcome
1.10E-09	0.081	0.004	0.009	0.677
2.58E-10	0.166	-0.002	0.006	0.803
2.23E-12	0.495	0.011	0.005	0.021
1.40E-12	0.175	0.001	0.006	0.861
2.85E-10	0.455	0.000	0.005	0.952
1.28E-15	0.023	-0.022	0.016	0.169
4.64E-16	0.209	0.007	0.006	0.271
2.56E-08	0.167	0.009	0.006	0.172
3.55E-13	0.357	0.003	0.005	0.540
3.10E-08	0.187	0.003	0.006	0.580
1.40E-16	0.218	-0.002	0.006	0.739
5.46E-30	0.781	0.000	0.006	0.994
9.43E-15	0.242	-0.004	0.006	0.518
1.47E-50	0.169	0.001	0.006	0.922
3.25E-26	0.101	-0.009	0.008	0.277
2.61E-49	0.472	-0.002	0.005	0.640
1.86E-10	0.527	-0.005	0.005	0.338
1.00E-200	0.104	0.001	0.008	0.922
8.13E-09	0.184	-0.002	0.006	0.697
3.15E-100	0.024	-0.004	0.016	0.794
3.78E-17	0.146	0.005	0.007	0.458
6.09E-09	0.841	-0.003	0.007	0.620
6.70E-13	0.926	-0.007	0.009	0.459
2.87E-08	0.261	-0.003	0.006	0.591
2.69E-08	0.172	-0.001	0.006	0.903
3.16E-08	0.134	0.005	0.007	0.444
8.53E-10	0.053	-0.011	0.011	0.303
5.40E-36	0.790	0.006	0.006	0.319
3.05E-08	0.322	-0.003	0.005	0.503
9.36E-14	0.374	-0.007	0.005	0.160
3.89E-08	0.098	-0.002	0.008	0.812
4.42E-08	0.073	0.000	0.009	0.981
1.79E-08	0.381	-0.002	0.005	0.734
4.24E-08	0.445	-0.011	0.005	0.027
6.83E-10	0.838	-0.001	0.007	0.896
8.38E-09	0.456	0.001	0.005	0.757
3.59E-12	0.753	-0.003	0.006	0.613
6.94E-10	0.451	0.009	0.005	0.054
6.88E-31	0.590	0.005	0.005	0.266
4.91E-08	0.544	-0.010	0.005	0.033
1.57E-32	0.164	0.000	0.006	0.957
7.59E-09	0.743	0.002	0.006	0.749
6.95E-12	0.413	-0.007	0.005	0.131
1.29E-24	0.608	0.003	0.005	0.587
2.87E-09	0.396	-0.005	0.005	0.332
3.46E-08	0.368	0.000	0.005	0.968
6.95E-10	0.768	-0.011	0.006	0.055
3.90E-10	0.172	0.009	0.006	0.153
3.32E-08	0.869	0.004	0.007	0.593
3.51E-28	0.852	-0.009	0.007	0.197
2.52E-20	0.105	0.011	0.008	0.149
2.53E-12	0.726	0.007	0.005	0.182
2.92E-09	0.792	-0.002	0.006	0.706
7.30E-15	0.462	-0.010	0.005	0.039
3.49E-08	0.517	-0.016	0.005	0.001
1.44E-08	0.046	-0.008	0.012	0.489

1.59E-10	0.593	0.003	0.005	0.478
2.15E-15	0.800	-0.002	0.006	0.706
7.24E-78	0.434	0.002	0.005	0.666
5.81E-18	0.091	0.011	0.008	0.209
8.15E-19	0.113	-0.007	0.008	0.341
1.05E-08	0.727	0.012	0.005	0.025
1.10E-20	0.746	-0.006	0.006	0.247
1.70E-10	0.580	0.010	0.005	0.051
1.34E-08	0.596	0.012	0.005	0.012
2.90E-10	0.347	0.009	0.005	0.080
7.59E-21	0.341	0.004	0.005	0.463
7.94E-10	0.164	-0.004	0.007	0.514
2.56E-08	0.520	0.006	0.005	0.210
